# Identification of a Second Type of AHL-lactonase from *Rhodococcus* sp. BH4, belonging to the α/β Hydrolase Superfamily

**DOI:** 10.4014/jmb.2001.01006

**Published:** 2020-03-09

**Authors:** Du-Hwan Ryu, Sang-Won Lee, Viktorija Mikolaityte, Yea-Won Kim, Haeyoung Jeong, Sang Jun Lee, Chung-Hak Lee, Jung-Kee Lee

**Affiliations:** 1 Department of Biomedicinal Science and Biotechnology, Paichai University, Daejeon 35345, Republic of Korea; 2 Infectious Disease Research Center, Korea Research Institute of Bioscience and Biotechnology (KRIBB), Daejeon 34141, Republic of Korea; 3 Department of Systems Biotechnology, Chung-Ang University, Anseong 17546, Republic of Korea; 4 School of Chemical and Biological Engineering, Seoul National University, Seoul 08826, Republic of Korea

**Keywords:** Quorum quenching, AHL, *Rhodococcus* spp., quorum sensing, AHL-lactonase, biofilm

## Abstract

*N*-acyl-homoserine lactone (AHL)-mediated quorum sensing (QS) plays a major role in development of biofilms, which contribute to rise in infections and biofouling in water-related industries. Interference in QS, called quorum quenching (QQ), has recieved a lot of attention in recent years. *Rhodococcus* spp. are known to have prominent quorum quenching activity and in previous reports it was suggested that this genus possesses multiple QQ enzymes, but only one gene, *qsdA*, which encodes an AHL-lactonase belonging to phosphotriesterase family, has been identified. Therefore, we conducted a whole genome sequencing and analysis of *Rhodococcus* sp. BH4 isolated from a wastewater treatment plant. The sequencing revealed another gene encoding a QQ enzyme (named *jydB*) that exhibited a high AHL degrading activity. This QQ enzyme had a 46% amino acid sequence similarity with the AHL-lactonase (AidH) of *Ochrobactrum* sp. T63. HPLC analysis and AHL restoration experiments by acidification revealed that the *jydB* gene encodes an AHL-lactonase which shares the known characteristics of the α/β hydrolase family. Purified recombinant JydB demonstrated a high hydrolytic activity against various AHLs. Kinetic analysis of JydB revealed a high catalytic efficiency (*k*_cat_/*K*_M_) against C4-HSL and 3-oxo-C6 HSL, ranging from 1.88 × 10^6^ to 1.45 × 10^6^ M^-1^ s^-1^, with distinctly low *K*_M_ values (0.16-0.24 mM). This study affirms that the AHL degrading activity and biofilm inhibition ability of *Rhodococcus* sp. BH4 may be due to the presence of multiple quorum quenching enzymes, including two types of AHL-lactonases, in addition to AHL-acylase and oxidoreductase, for which the genes have yet to be described.

## Introduction

Many bacteria can control the expression of diverse genes in response to cell density via the quorum sensing (QS) system. In particular, Gram-negative bacteria use *N*-acyl-homoserine lactones (AHLs) as signaling molecules to induce or repress expression of specific phenotypes [1, 2]. Characteristic of typical group behavior, biofilm formation is also controlled by QS, and extensive studies about the relationship between QS and biofilm formation have been reported [3, 4]. Biofilm is one of the main virulence factors in many pathogenic bacteria and the cause of biofouling in water-related industrial systems [4, 5]. As a result, QS is an attractive target for anti-biofilm and anti-biofouling approaches, and there are several quorum quenching techniques used to disrupt the quorum sensing system [2, 6]. Among them, enzymatic degradation or modification of AHL signaling molecules is an efficient way to disrupt AHL-based QS [7-10]. To date several diverse QQ enzymes have been identified, which can be broadly classified into three types [10]. AHL-lactonases open the homoserine lactone ring [7, 8], AHL-acylases (also referred as amidohydrolases) cleave the amide bond between the acyl chain and the homoserine lactone ring, releasing fatty acid and homoserine lactone [11, 12]. AHL-oxidoreductases are modifying enzymes divided into two groups; reductases can convert 3-oxo-substituted AHLs to 3-hydroxyl AHLs, and cytochrome oxidases catalyze oxidation of the acyl chain [9, 13, 14]. Thus, the resulting modified compounds can no longer function as signal molecules.

AHL-lactonases have been the most extensively studied type of QQ enzyme. They have been divided into four different classes based on their amino acid sequences and structures; the metallo-β-lactamase (MBL) superfamily, the phosphotriesterase (PTE) family, the α/β hydrolase family, and the GDSL-like hydrolase family [2,5,15-17]. A lactonase from the *Bacillus* sp. strain 240B1, AiiA, was the first reported QQ enzyme [18], which belongs to the most abundant and extensively studied metallo-β-lactamase superfamily [19]. Afterwards, the other families of AHL-lactonase enzymes were identified with QsdA of *Rhodococcus erythropolis* W2 being the first reported case for the PTE family lactonase [20]. The MBL and PTE family lactonases are metal-dependent proteins [5, 20, 21]. In contrast, recently identified α/β hydrolase family lactonases, AiiM from *Microbacterim testaceum* StL B037 and AidH from *Ochrobactrum* sp. strain T63, do not have a metal binding motif, indicating an entirely different AHL hydrolyzing mechanism than those observed in MBL and PTE families [5, 22, 23].

The genus *Rhodococcus* is a member of the Gram-positive actinobacteria phylum, which possesses a high G+C content and are typically isolated from soil and water environments [24]. *Rhodococcus* strains have wide metabolic versatility and exhibit a remarkable ability to degrade organic and xenobiotic compounds, many of which are toxic [25]. Therefore, *Rhodococcus* have been used for environmental and biotechnological applications such as bioremediation and bioconversion [26]. Members of the *Rhodococcus* genus also have prominent quorum quenching ability and have been used for wastewater treatment and biocontrol [27, 28]. Recently, *Rhodococcus* sp. BH4, which was isolated by our group, has been applied for use in the reduction of biofouling in membrane bioreactors (MBR) utilized for advanced wastewater treatment [27]. Although, many bacteria have only one type of quorum quenching enzyme, *Rhodococcus* spp. have been reported to possess activities of AHL-lactonase, acylase and oxidoreductase for AHL signal inactivation [9, 20, 29]. However, despite the reputation of *Rhodococcus* spp. for encoding diverse QQ enzymes, only a PTE family AHL-lactonase (QsdA) has been reported [20]. In this study, we have identified the presence of another AHL-lactonase, which belongs to α/β hydrolase family, exhibiting a high AHL degrading activity and prominent biofilm inhibition capacity.

## Materials and Methods

### Bacterial Strains and Culture Media

*Chromobacterium violaceum* CV026, *Agrobacterium tumefaciens* NT1 (pDCI41E33) and *Agrobacterium tumefaciens* A136 (pCF218 and pCF372) were used as reporter strains for the detection of *N*-acyl-homoserine lactones [30-33]. *A. tumefaciens* NT1 was grown in AB minimal medium supplemented with 100 μg/ml carbenicillin at 30°C [31]. *A. tumefaciens* A136 was grown on LB agar with 4.5 μg/ml tetracycline and 50 μg/ml spectinomycin. For X-gal assay *A. tumefaciens* A136 was grown at 30°C in AT minimal medium containing 0.5%(wt/vol) glucose [33]. *Rhodococcus* sp. BH4 (KCTC 33122), biofilm producer *Aeromonas* sp. T3-4 and biosensor *C. violaceum* CV026 were cultivated in an LB medium at 30°C [27, 34]. Recombinant *E. coli* were maintained on LB medium supplemented with 50 μg/ml of kanamycin.

### Genome Sequencing, Analysis and Accession Numbers

The genome sequencing of *Rhodococcus* sp. BH4 was carried out at the National Instrumentation Center for Environmental Management in Seoul National University (Republic of Korea) using the PacBio RSII platform with P6-C4 chemistry (Pacific Biosciences, USA). RS_HGAP_Assembly.2 protocol under a SMRT Analysis version 2.3.0 environment was used for hierarchical genome assembly and polishing. Assembled sequences were manually circularized by inspecting overlapping sequence at the both ends of contig, followed by the adjustment of the start position of the chromosome using the *dnaA* gene sequence as the first gene closest to the origin of replication, and was later confirmed using Circlator (PMID 26714481). Annotation of the finalized genome sequence was performed with the Classic RAST pipeline on the RAST server (PMID 24293654) and with the NCBI Prokaryotic Genome Annotation Pipeline (PMID 27342282), the latter being used for the submission of the annotated genome sequence to GenBank. Genomic islands were predicted using IslandViewer version 4 (PMID 28472413). Multiple alignment of the completely sequenced chromosomes of related strains was carried out using Mauve version 2.4.0 (PMID 15231754). The genome sequence of *Rhocococcus* sp. BH4 was deposited in the GenBank database under accession numbers CP014941.1 (chromosome) and CP014942.1 (plasmid). Additionally, amino acid sequences of the QQ enzymes from *Rhodococcus* sp. BH4 can be found in the GenBank database under the following accession numbers; ARE36427.1 (QsdA) and ARE36482.1 (JydB).

### Cloning, Expression and Purification of JydB

Five genes encoding putative QQ enzymes in *Rhodococcus* sp. BH4 were cloned into pMD20-T vector and transformed into *E. coli* DH5α. A modified In-Fusion cloning method was used to construct the pET28a vector for the expression of newly identified AHL-lactonase (JydB) [35]. Primers used in this study are shown in Table S1. pET28a-*jydB* was transformed into *E. coli* BL21(DE3) and cultured until OD_600_ of 0.5 at 37°C with shaking at 200 rpm. Protein expression was induced with 0.1 mM isopropyl β-D-1-thiogalactopyranoside (IPTG) for 24 h at 16°C. After incubation, cultures were harvested by centrifugation (3,000 ×g, 4°C, 20 min) and resuspended in 10 ml of resuspension buffer (30mM imidazole, 50mM Tris-HCl, 1% glycerol and 300mM NaCl, pH 7.5). Suspension mixture was then sonicated on ice and centrifuged for 20min to remove cell debris. Protein was purified from the supernatant using a Ni-NTA column (QIAGEN, Germany). The His-tagged JydB protein was washed with washing buffer (30mM imidazole, 50mM Tris-HCl, 1% glycerol and 300mM NaCl, pH 7.5) and eluted using elution buffer (250mM imidazole, 50mM Tris-HCl, 1% glycerol and 300mM NaCl, pH 7.5). The purity and size of the protein were confirmed by SDS-PAGE. Concentration was estimated using the Bradford assay.

### Bioassay of AHL Degrading Activity

The *N*-acyl-homoserine lactones used in this study were; *N*-butyryl-l-homoserine lactone (C4-HSL), *N*-hexanoyl-l-homoserine lactone (C6-HSL), *N*-octanoyl-l-homoserine lactone (C8-HSL), *N*-decanoyl-l-homoserine lactone (C10-HSL), *N*-dodecanoyl-l-homoserine lactone (C12-HSL), *N*-(3-oxohexanoyl)-l-homoserine lactone (3-oxo-C6-HSL), *N*-(3-oxooctanoyl)-l-homoserine lactone (3-oxo-C8-HSL) and *N*-(3-oxododecanoyl)-l-homoserine lactone (3-oxo-C12-HSL), which were purchased from BNPharm Co, Ltd (Daejeon, South Korea). In order to determine the activity of JydB, 6 μg of purified enzyme was mixed with AHLs (final concentrations depended on the biosensor: 20 μM for CV026, 5 μM and 1 μM for NT1 and 1 μM for A136) in Tris-HCl buffer (10 mM pH 7.0) and incubated at 37°C for 20 min, with shaking. After boiling for 5 min to stop the reaction, 20 μl of reaction mixtures were loaded into the wells of the reporter strain overlaid agar plates or 10 μl of reaction mixtures were mixed with 190 μl of A136 X-gal assay solution (1% of A136 culture was inoculated into AT media containing 150 μg/ml of X-gal) in 96-well plate [33, 36]. Samples containing known concentrations of AHLs were used as the control. Controls were incubated and boiled together with the reaction mixture samples.

### Kinetic Analysis of AHL-lactonase

For kinetic analysis, enzyme activity was measured spectrophotometrically. Proton release from the hydrolysis of the AHL lactone ring was measured in weakly buffered solutions using the pH sensitive dye, phenol red. The reaction mixture contained 50 μM phenol red (pH 7.5), 200 mM NaCl, 1 mM HEPES, 0 to 4 mM AHL substrates (C4-HSL, C6-HSL and 3-oxo-C6-HSL) and 6 μg of the purified JydB. Hydrolysis was measured by monitoring the decrease in A_557_ over time using microplate reader (VersaMax, Molecular Devices Inc., USA) [37].

### HPLC Analysis and AHL Restoration by Acidification

High-performance liquid chromatography (HPLC) analysis was carried out to analyze AHL degradation products. For the hydrolysis assay, reaction mixture containing 4.6 μg of purified enzyme and 1 mM C6-HSL was incubation at 37°C for 15 min. Reactions were stopped by boiling and the mixture was centrifuged to pellet the precipitated protein. Samples were chromatographed on an HPLC system with a UV/visible light (VIS) detector set at 205 nm by use of a ZORBAX Eclipse XDB-C18 column (4.6 × 250 mm) (Agilent Technologies). Samples were then eluted isocratically with water-acetonitrile-acetic acid (74.75:25:0.25 [vol/vol/vol]) at a flow rate of 1 ml/min [38]. AHL degradation product was observed by comparing the reduction in the peak areas for a given retention time with samples containing a known concentration of C6-HSL. For AHL restoration experiments, samples from AHL degradation assays were divided into aliquots. One of each was acidified with 1N HCl to cause restoration of the lactone ring which was opened by AHL-lactonase activity. The acidified sample was incubated at 4°C for 24 h and then loaded into the wells of biosensor overlaid plates [39].

### Inhibition of Biofilm Formation

In order to examine inhibition of biofilm formation by JydB, a static microtiter plate assay was carried out by methods previously described [40]. Biofilm producing *Aeromonas* sp. T3-4 was incubated for 12 h with 17 μg of purified JydB. Turbidity was measured at OD_600_ following the incubation. Afterwards, planktonic cells were removed and washed with buffer (10 mM Tris-HCl pH 7.0). Biofilms were detected by staining with 0.1% crystal violet for 30 min at room temperature and then washing thoroughly with Tris-HCl buffer. For the quantitative analysis of biofilm formation, 200 μl ethanol (95%) was used to destain the wells, and absorbance was measured at 550 nm using a microplate reader (VersaMax, Molecular Devices Inc., USA).

### Statistical Analysis

All data are shown as mean ± the standard deviation. The one-tailed Student’s *t*-test was used to assess significant difference between groups. *P*-values of less than 0.05 were considered as significant.

## Results

### Genome Properties of *Rhodococcus* sp. BH4

Previously, we isolated *Rhodococcus* sp. BH4 from a wastewater treatment facility and it exhibited not only AHL degrading activity, but also effectively inhibited biofouling in the MBR [27]. To further analyze the strain, *Rhodococcus* sp. BH4 genome was sequenced using the PacBio RSII platform. *De novo* assembly of 72,529 long reads (849,949,298 bp total, *N*_50_ of 16,008 bp) using HGAP yielded two contigs. After processing, the circular chromosome and one putative linear plasmid, 6,314,891 and 704,258 bp in respective size, were obtained. The general features of the genome of the BH4 strain are listed in Table 1.

### Analysis of the Sequenced Genome and Cloning of Genes Encoding Putative QQ Enzymes

**Fig. 1:**
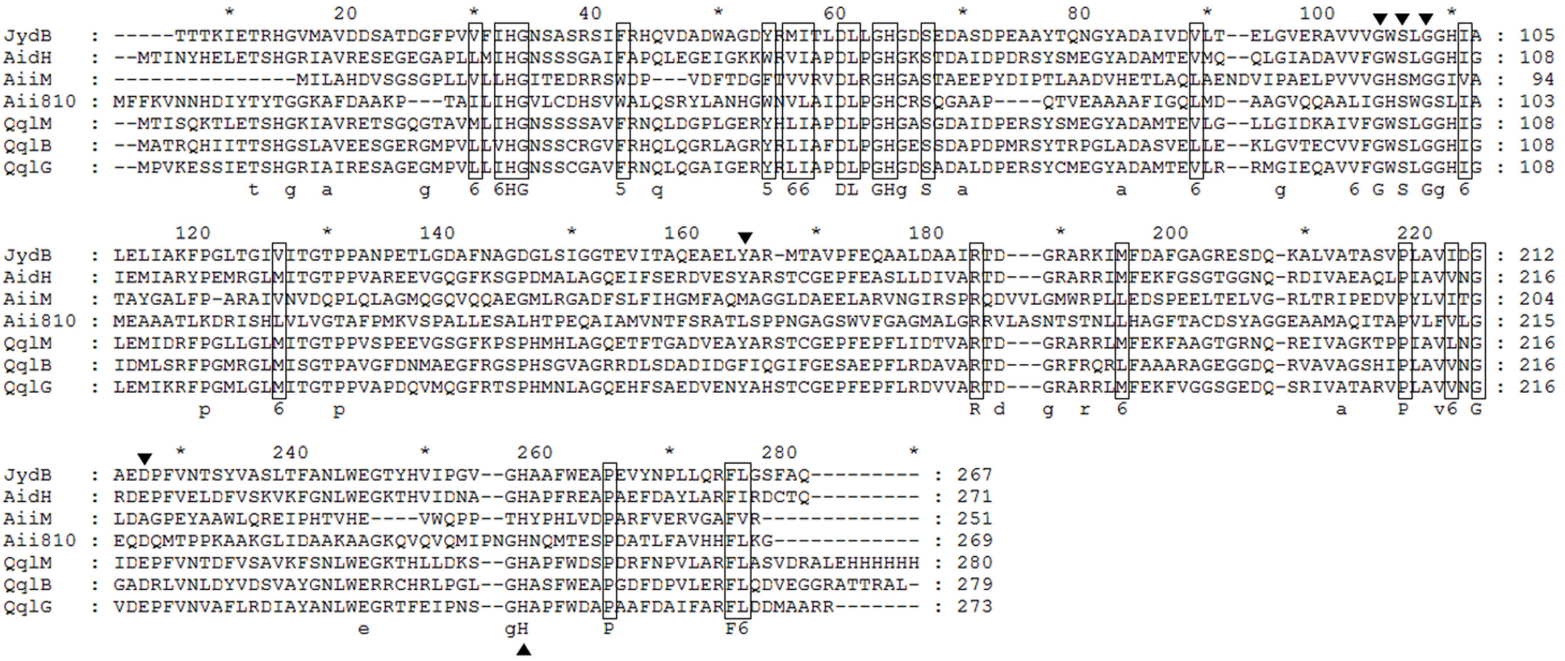
Amino acid sequence alignment of JydB with other α/β-hydrolases. Aligned amino acid sequences are for the following enzymes; JydB (ARE36482) from *Rhodococcus* sp. BH4 with AidH from *Ochrobactrum* sp. T63 (ACZ73823) (46%), QqlM from *Mesorhizobium cicero* (AMQ81197) (47%), Aii810 from Mao-tofu metagenome (ASY06633) (28%), AiiM from Microbacterium testaceum (BAK74763) (31%), QqlB from *Paraburkholderia glathei* (AMQ81195) (44%), and QqlG from *Geminicoccus roseus* (AMQ81196) (47%). Amino acid sequence identities of JydB with other AHL-lactonases are shown in parentheses. The alignment was generated by the ClustalO program. All highly conserved amino acid residues are marked with rectangles. The putative catalytic triad amino acid residues essential for AHL degrading activity are indicated with triangles.

Even though there are many reports concerning the presence of diverse QQ enzymes in some *Rhodococcus* spp., only one AHL-lactonase gene (*qsdA*) has been identified [20, 29]. Therefore, genome analysis of *Rhodococcus* sp. BH4 was carried out to explore other putative genes encoding QQ enzymes using BLASTP (Basic Local Alignment Search Tool Program). Five candidate genes for the QQ enzymes were amplified by PCR, besides the *qsdA* gene (A0W34_26390) which possesses a 99% amino acid sequence identity with QsdA from the *Rhodococcus erythropolis* strain W2 [20]. These five genes (A0W34_26705, A0W34_24065, A0W34_00835, A0W34_31420, A0W34_03910) shared a 35%-45% amino acid sequence identity with sequences of known AHL-lactonases, AHL-acylases, and oxidoreductases (Table S2). These genes were cloned into pMD20 T vector and expressed in *E. coli* DH5α (Fig. S1). Only recombinant *E. coli* containing the A0W34_26705 gene demonstrated AHL degrading activity (Table S2). This gene encodes a predicted protein of 268 amino acid residues (named JydB), which possessed an amino acid sequence identity of 46%-47% with AHL-lactonases AidH, QqlG, and QqlM from *Ochrobactrum* sp. T63, *Geminicoccus roseus*, and *Mesorhizobium ciceri*, respectively [22, 38]. However, it exhibited a relatively low similarity to other AHL-lactonases such as, AiiM from *Microbacterim testaceum* StL B037 (31%), and AiiA810 from the Mao-tofu metagenome (28%) [23, 41] (Fig. 1). Multiple sequence alignment of JydB and other known AHL-lactonases revealed that JydB shares many known characteristics with proteins from the α/β hydrolase family [16, 42]. The typical conserved catalytic triad of active site serine (S100) of the G-X-S-X-G motif, aspartic acid (D216), and histidine (H245) residues were found in the JydB protein (Fig. 1). Gao *et al*. suggested that Tyr160 of AidH, a representative of the α/β hydrolase family AHL-lactonase, is important for biocatalysis based on its crystalline structure [43]. The corresponding tyrosine residue (Tyr158) is also conserved in JydB. Therefore, we concluded that *Rhodococcus* sp. BH4 also has an AHL-lactonase that belongs to the α/β hydrolase family, besides the previously reported QsdA, which is a phosphotriesterase (PTE) family member.

### AHL Hydrolytic Activity of JydB and AHL Restoration by Acidification

**Fig. 2:**
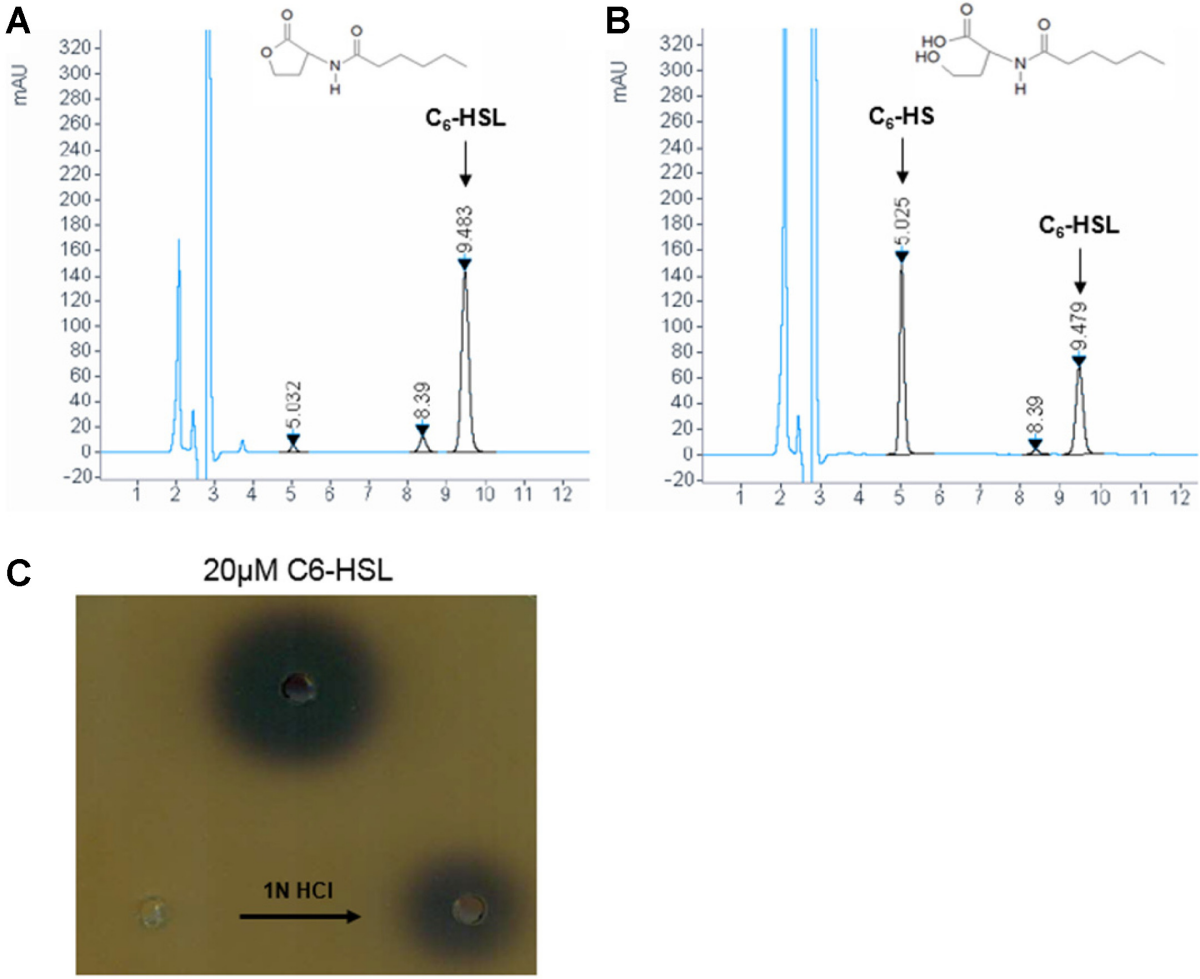
HPLC analysis of C6-HSL degradation by JydB and restoration of C6-HSL by acidification of JydBhydrolysis product. (**A**) 1 mM C6-HSL substrate peak is shown as the control with an elution time of 9.5 min, which was monitored at 205 nm. (**B**) C6-HSL hydrolysis reaction was carried out with 4.6 μg JydB and 1 mM C6-HSL at 37°C for 15 min and terminated by boiling. The profile of the JydB-hydrolyzed C6-HSL product shows a new peak (5 min) of hydrolyzed product, whereas the C6-HSL substrate peak (9.5 min) is decreased. (**C**) C6-HSL was restored by acidification of JydBhydrolyzed C6-HSL product. The reaction mixture was acidified to pH 2 by adding 1N HCl and incubated at 4°C for 24 h.

A modified In-Fusion cloning method was used to construct the pET28a-*jydB* vector for the expression of the His-tagged protein. The expression of *jydB* gene in recombinant *E. coli* was induced by IPTG, and then the expressed soluble protein was purified using a Ni-NTA column. The size of the purified *N*- and C-terminal His-tagged recombinant protein was estimated at around 31 kDa by SDS PAGE (Fig. S2), which corresponded with the predicted molecular weight of 28.4 kDa. The purified His-tagged JydB was used to assess the activity and catalytic mechanism of the protein. Activity of the purified protein was confirmed using bioreporter system against C6-HSL, 3-oxo-C6-HSL and C8-HSL (Fig. S3). Afterwards, HPLC was carried out to determine hydrolytic activity of JydB. C6-HSL was selected as a substrate because it was initially used for isolation of *Rhodococcus* sp. BH4 [27]. Fig. 2A shows intact C6-HSL with a retention time of 9.5 min as the control. In Fig. 2B a reaction product with a retention time of 5 min was produced by the enzymatic digestion of C6-HSL by JydB, which was accompanied by decrease in a C6-HSL peak at 5 min when compared to the control sample (shown in Fig. 2A). Even though AHL-lactonase (JydB) of *Rhodococcus* sp. BH4 possesses highly conserved catalytic site of α/β hydrolase family, it also has a 45% amino acid sequence identity with AHL-acylase, AiiO, from *Ochrobactrum* sp. A44 [44]. Therefore, to determine whether this QQ gene encodes AHL-lactonase or acylase, an acidification experiment of the reaction product was conducted in addition to HPLC. In the case of AHL-lactonase reactions, the enzyme opens the ring structure of the *N*-acyl-homoserine lactone, which can be relactonized by acidification with HCl [39]. Biosensor CV026 was used to examine whether the AHL could be restored by acidification of the reaction mixture. Bioassays revealed that JydB can degrade 1mM C6-HSL within 20 min at 37°C, and the AHLs degraded by the recombinant JydB were restored after acidification (Fig. 2C), supporting the hypothesis that the catalytic mechanism of JydB involves cleavage of the lactone ring of AHL. Therefore, the degradation product of C6-HSL (5 min peak), which appeared in HPLC, is considered to be the C6-HS peak. Amino acid sequence analysis of JydB, HPLC results and the restoration of AHLs by acidification prove that the cloned *jydB* gene encodes an AHL-lactonase.

**Fig. 3:**
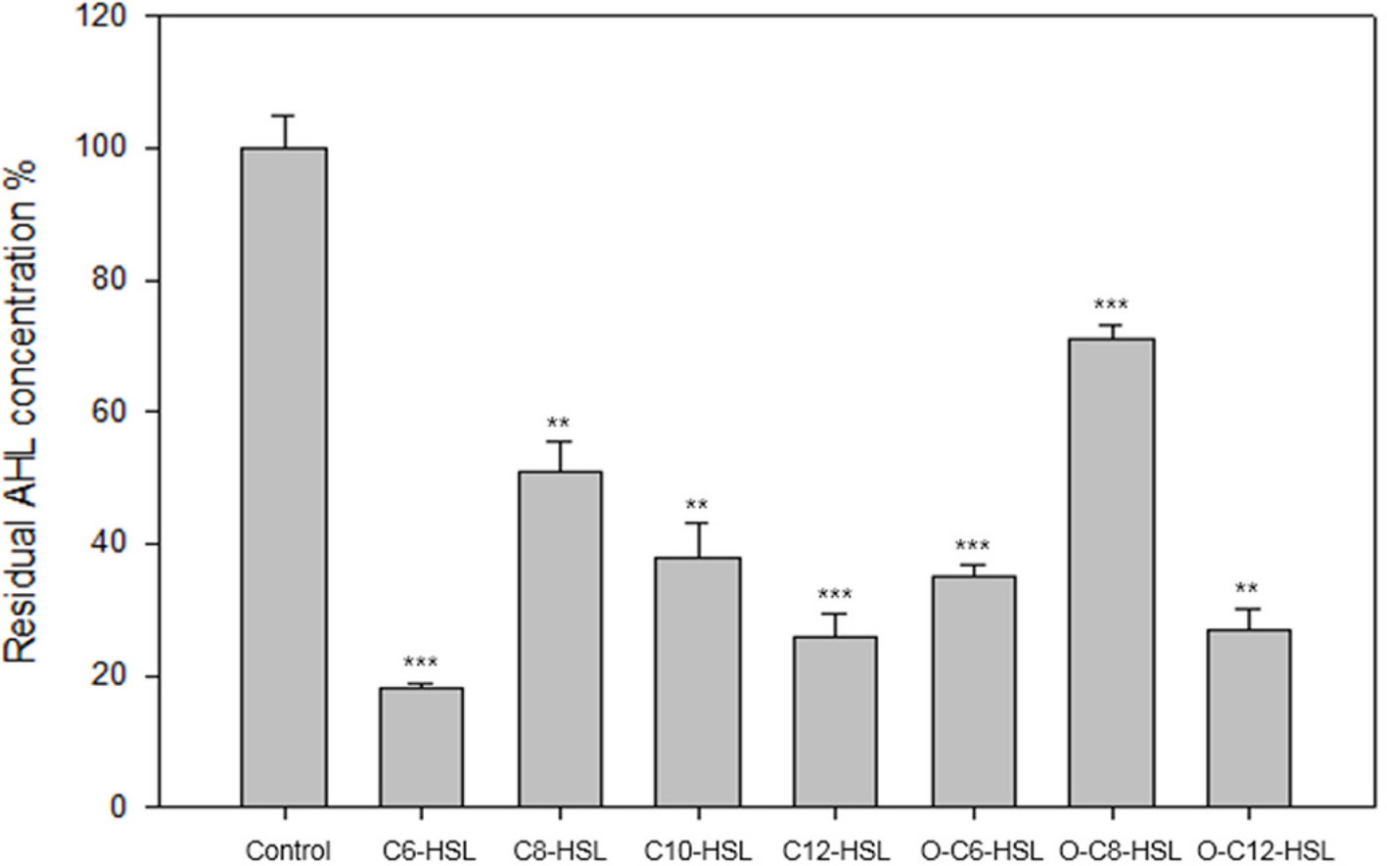
AHL-degrading activity of JydB using various AHL substrates. Six μg/ml of purified protein was mixed with 1 μM of different AHLs and incubated for 5 min at 37°C. Residual AHL concentration was detected using *Agrobacterium tumefaciens* A136 bioassay system by measuring β-galactosidase activity spectrophotometrically. The average of values obtained from spectrophotometric measurement of all 1 μM AHL control samples is depicted as the relative control in the figure. Data represent the mean of the triplicate measurements ± the standard deviation. **p < 0.01. ***p < 0.001.

Hydrolytic activity of JydB was also analyzed using different AHLs, to examine if JydB has substrate preference for AHLs of certain lengths or oxo-substitutions. Fig. 3 shows residual AHL concentration after reactions with JydB using a bioassay strain, *A. tumefaciens* A136. JydB showed broad substrate specificity, efficiently degrading 60-80% of most of the AHL substrates within 5min, however a relatively reduced activity was seen against C8-HSL and 3-oxo-C8-HSL (Fig. 3).

### Kinetic Analysis of Recombinant AHL-lactonase JydB

Kinetic analysis was carried out to characterize the purified JydB using phenol red, a pH indicator, by monitoring the release of H^+^ during the enzymatic degradation of C4-HSL, C6-HSL, and oxo-C6-HSL. Lineweaver-Burk plot was used to determine the kinetic constants (*K*_M_ and *k*_cat_). JydB hydrolyzed AHLs very efficiently with *K*_M_ values ranging from 0.66 to 15 mM and *k*_cat_/*K*_M_ values ranging from 4.36 × 10^4^ to 1.88 × 10^6^ s^-1^M^-1^ (Table 2). In particular, JydB showed a high affinity for C4-HSL and 3-oxo-C6-HSL with *K*_M_ values of 0.16 mM and 0.24 mM, respectively. The *k*_cat_/*K*_M_ value for C4-HSL and 3-oxo-C6-HSL was 43- and 33-fold higher than that of C6-HSL.

### Inhibition of Biofilm Formation by Recombinant JydB

**Fig. 4:**
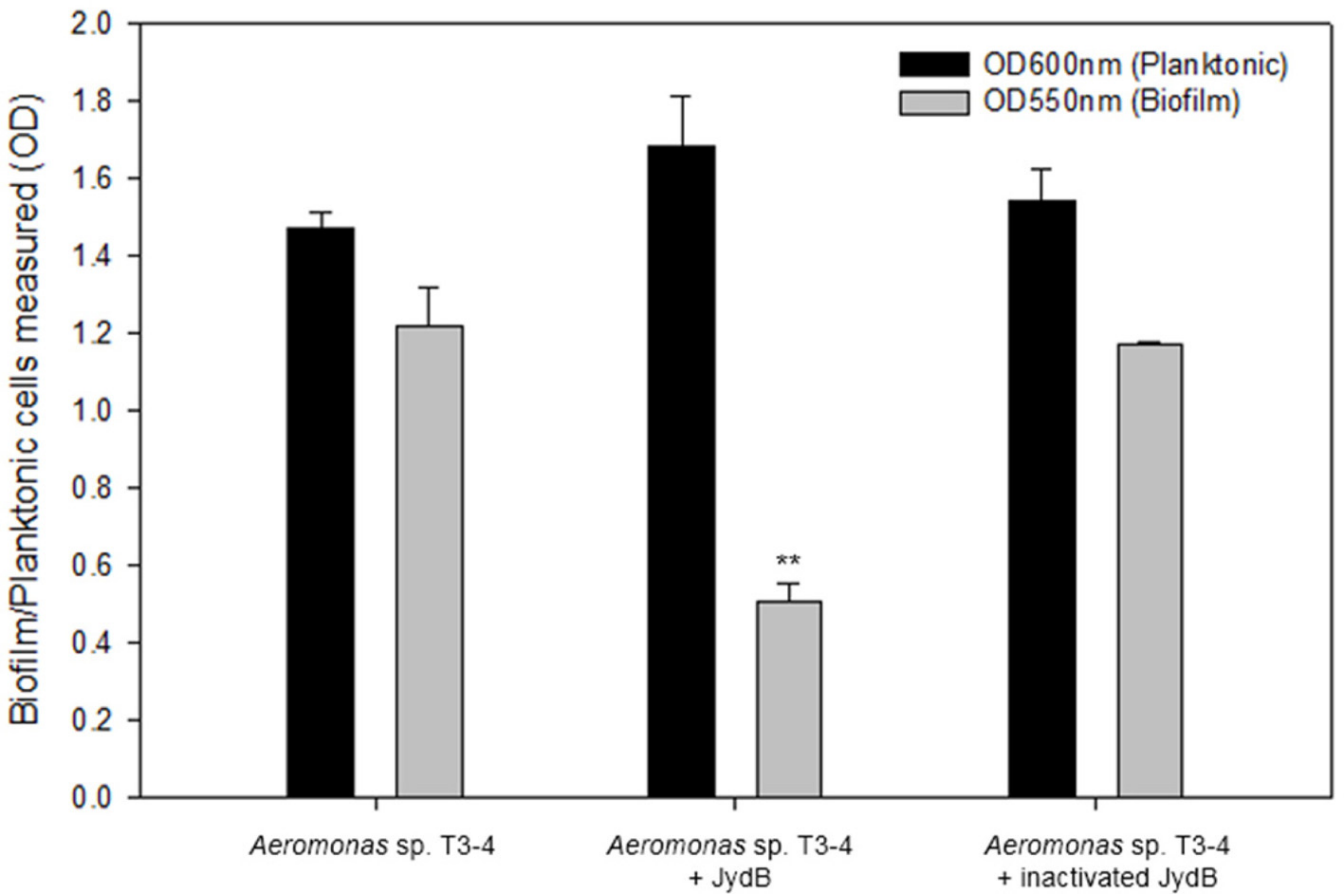
Inhibition of biofilm development using 96-well microtiter plate. Seventeen μg of purified JydB and *Aeromonas* sp. T3-4 were cultivated together in a 96-well microtiter plate for 12 h. Turbidity was measured at OD_600_ prior the treatment with 0.1% crystal violet (black bars). Formation of biofilm was measured at OD_550_ (gray bars). Heat inactivated JydB was used as control. All experiments were carried out in triplicates and data were exhibited as the mean ± the standard deviation. **p < 0.01.

To investigate whether recombinant JydB has the ability to inhibit biofilm formation, static microtiter plate assay, and a higher scale assay using a slide glass (data not shown), were carried out using biofilm producer *Aeromonas* sp. T3-4 [34]. *Aeromonas* sp. T3-4 was incubated for 12hr with purified JydB to determine the effect on biofilm formation. Fig. 4 shows that JydB can significantly reduce biofilm formation without inhibiting the growth of *Aeromonas* sp. T3-4, because the OD_600_ remained similar in the control and samples containing the enzyme, as seen in Fig 4. These results suggest that the recombinant AHL-lactonase JydB is able to inhibit biofilm development by *Aeromonas* sp. T3-4 via AHL degradation.

## Discussion

**Fig. 5:**
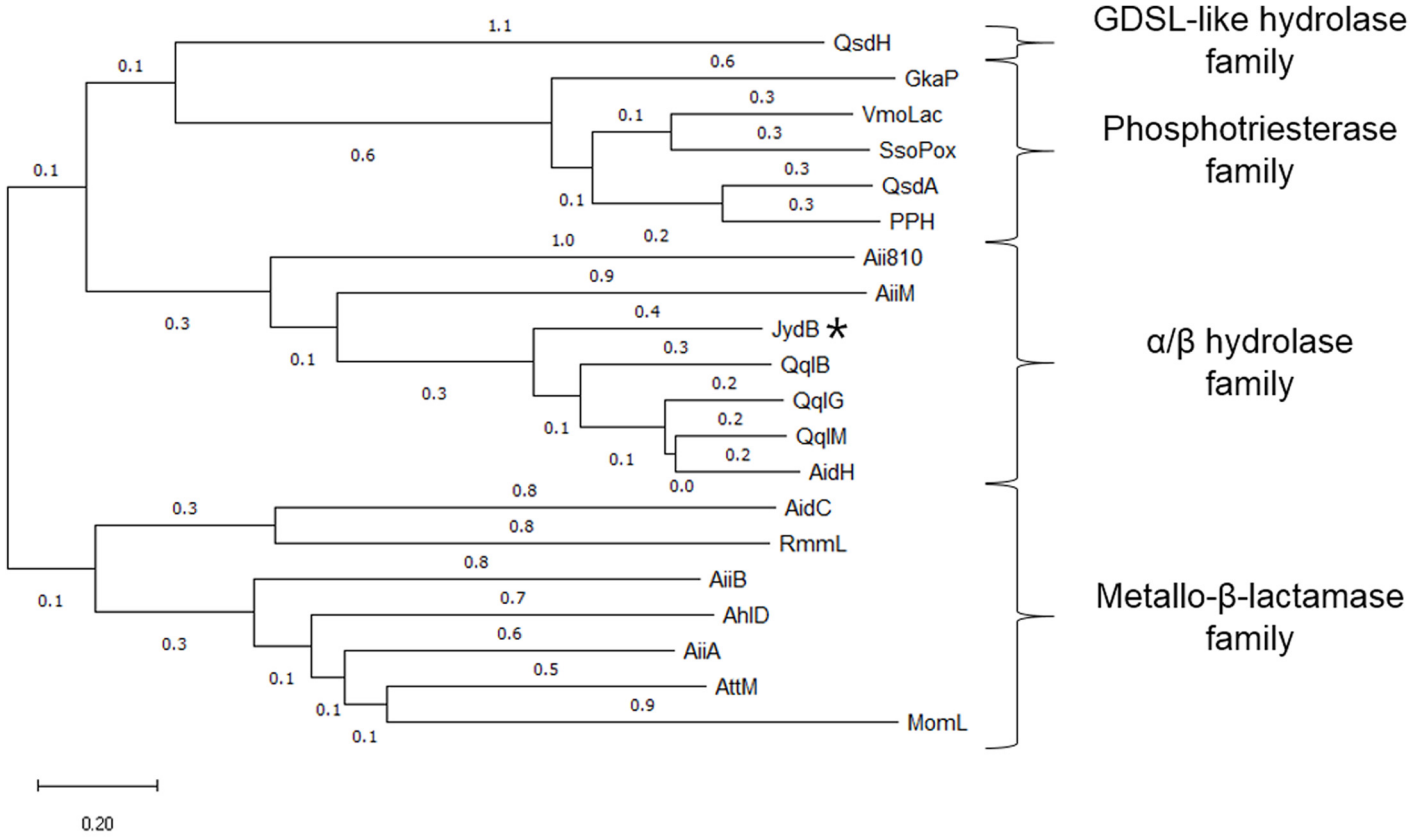
Phylogenetic analysis of JydB and other known AHL-lactonases. Sequences used for the analysis were; AhlD from *Arthrobacter* sp. IBN110, AiiA from *Bacillus* sp. A24, RmmL from *Ruegeria mobilis* YJ3, AiiB from *Agrobacterium tumefaciens*, AttM from *Agrobacterium tumefaciens*, AidC from *Chryseobacterium* sp. StRB126, MomL from *Muricauda olearia*, QsdA from *Rhodococcus erythropolis* W2, AidH from *Ochrobactrum* sp. T63, AiiM from *Microbacterium testaceum*, QsdH from *Pseudoalteromonas byunsanensis*, GkaP from *Geobacillus kaustophilus*, VmoLac from *Vulcanisaeta moutnovskia*, SsoPox from *Sulfolobus solfataricus*, Aii810 from Mao-tofu metagenome, QqlB from *Paraburkholderia glathei*, QqlM from *Mesorhizobium ciceri*, and QqlG from *Geminicoccus roseus*. JydB is indicated with an asterisk. The dendrogram was constructed using the neighbor-joining method with MEGA X software (http://www.megasoftware.net/). The scale bar represents 0.2 substitutions per amino acid position.

More than 30 AHL-lactonases have been identified experimentally, and four different AHL-lactonase families (MBL, PTE, α/β hydrolase, and GDSL-like hydrolase) have been described [16]. Although many bacteria have a single type of quorum quenching enzyme, some strains encoding multiple QQ enzymes were recently reported [9, 16, 45, 46]. Several lactonases belonging to the MBL family were found in the *Acinetobacter baumannii* ATCC 17978 [45]. The *Rhizobium* sp. strain NGR234 has five QQ enzymes, including two lactonases DhlR and QsdR1 [16], and Pseudomonas aeruginosa PAO1 possesses three AHL-acylases (PvdQ, QuiP, and HecB) that belong to the Ntn hydrolase superfamily [9, 46]. *Rhodococcus* spp. were also reported to show activities for diverse QQ enzymes including AHL-lactonase, acylase, and oxidoreductase. However, despite the reputation of *Rhodococcus* spp. as the reservoir of QQ enzymes [9, 29], only a single *qsdA* lactonase gene has been reported to date [20]. In this study, we have identified another AHL-lactonase in *Rhodococcus* sp. BH4, which belongs to the α/β hydrolase family. Phylogenetic analysis of JydB and other AHL-lactonases from various bacteria is shown in Fig. 5. At this point in time, only a limited number of α/β hydrolase superfamily AHL-lactonases, including AidH from *Ochrobactrum* sp. T63, AiiM from *Microbacterim testaceum* StL B037 and AiiA810 from the Mao-tofu metagenome, QqlG from *Geminicoccus roseus*, QqlM from *Mesorhizobium cicero*, and QqlB from *Paraburkholderia glathei*, besides JydB, have been identified experimentally [22, 38, 47, 48]. As shown in Fig. 1, JydB has a relatively low similarity with other AHL-lactonases belonging to the same α/β hydrolase family, but the catalytic site is highly conseved. Therefore, cloned JydB can be added to the list for the α/β hydrolase cluster.

Kinetic analysis of JydB revealed a high catalytic efficiency against short chained AHLs (C4-HSL) and AHLs with 3-oxo side chain (3-oxo-C6-HSL), and the *k*_cat_/*K*_M_ values ranged from 1.88 × 10^6^ to 1.45 × 10^6^ M^-1^ s^-1^, with distinctly low *K*_M_ values (0.16 - 0.24 mM). Afriat *et al*. [49] reported that QsdA exhibited catalytic efficiency (*k*_cat_/*K*_M_) of 1.5 × 10^5^ M^-1^ s^-1^ towards C4-HSL, which is about 12-fold lower than that of JydB. Additionally, we tested the hydrolytic activity of JydB against AHLs of various lengths and oxo-substitutions using *A. tumefaciens* A136 biosensor. JydB efficiently hydrolyzed majority of AHLs regardless of length and oxo-substitutions. Accordingly, JydB has a broad acyl chain length spectrum like AidH of *Ochrobactrum* sp. T63. Similarly to the PTE family, AHL-lactonase QsdA from *R. erythropolis* strain W2 was also reported to have broad substrate spectrum [20]. The high activity and broad substrate spectrum of JydB implicate that it might be a major QQ enzyme among several others present in *Rhodococcus* sp. BH4, and it could be used for practical applications.

*Rhodococcus* spp. have important biotechnological and environmental aspects because of their robustness and broad catalytic diversity to degrade a wide range of organic compounds including xenobiotics [24-26]. *Rhodococcus* sp. BH4 shows high AHL degrading activity and biofilm inhibition capacity compared to other QQ strains [27, 34]. We think that the prominent AHL degrading activity of *Rhodococcus* sp. BH4 is caused by multiple QQ enzymes, including two types of AHL-lactonases and putative AHL-acylase and oxidoreductase. Additional studies are required to identify corresponding genes for other QQ enzymes in *Rhodococcus* sp. BH4.

Besides AHL degradation, other cellular roles of the two AHL degrading enzymes in *Rhodococcus* sp BH4 might be present. AHL-lactonases belonging to the α/β hydrolase family, including JydB, share the typical catalytic triad residues (serine, aspartic acid, and histidine) with some lipases and esterases [45, 48]. Thus, JydB is also likely to exhibit a broad substrate spectrum. On the other hand, PTE-like lactonases (PLLs) like QsdA from *Rhodococcus erythropolis* have phosphotriesterase activity which is involved in the hydrolysis of organophosphates such as paraoxon, a synthetic pesticide [49]. It would be interesting not to only develop *Rhodococcus* as a potent QQ strain that interferes with QS, but also to elucidate the other physiological and ecological roles of these two AHL-lactonases in *Rhodococcus* sp. BH4.

## Supplemental Materials

Supplementary data for this paper are available on-line only at http://jmb.or.kr.

## Figures and Tables

**Table 1:** General features of *Rhodococcus* sp. BH4 genome.

Genome features	Value
Chromosome	6,314,891 bp
Plasmid	704,258 bp
G+C content	62.3%
rRNA (5S, 16S, 23S)	15 (5, 5, 5)
tRNA	53
Number of coding sequences	6,342

**Table 2:** Kinetic constants of AHL-lactonase JydB for hydrolysis of AHLs.

Substrates	*k*_cat_ (s^-1^)	*K*_M_ (mM)	*k*cat/*K*m (M^-1^ s^-1^)
C_4_-HSL	300 ± 17.32	0.16 ± 0.031	(1.88 ± 0.31) × 10^6^
C_6_-HSL	666.67 ± 50.92	15.28 ± 2.41	(4.36 ± 0.11) × 10^4^
3-oxo-C_6_-HSL	347.32 ± 24.73	0.24 ± 0.07	(1.45 ± 0.39) × 10^6^
