# Supplementary material for: Identification of a Second Type of AHL-lactonase from Rhodococcus sp. BH4, belonging to the α/β Hydrolase Superfamily
Source: J Microbiol Biotechnol. 2020 Mar 9;30(6):937–45. doi: 10.4014/jmb.2001.01006 (PMC9728292; doi:10.4014/jmb.2001.01006)

J. Microbiol. Biotechnol.
https://doi.org/10.4014/jmb.2001.01006

Table S1 Primers used in this study

Primer name	Sequence	Use in this work
A0W34_26705 (A1)-F	5'-ATCTCGAATTCAGAGCGCGGAACATTCTCT-3'	Amplification of gene fragments of putative QQ enzymes from *Rhodococcus* sp. BH4
A0W34_26705 (A1)-R	5'-ATCGGTACCTTTCCGAACTGTAGGACGG-3'	
A0W34_24065 (A2-1)-F	5'-ATCTCGAATTCCGCCGAAACCTTCCGCG-3'	
A0W34_24065 (A2-1)-R	5'-ATCGGTACCGAGTACCTTCTCAGCCAAG-3'	
A0W34_00835 (A2-2)-F	5'-ATATCGAATTCTCGTTGAAACTCGTCAGAAC-3'	
A0W34_00835 (A2-2)-R	5'-ATCCCCGGGGCCACCACGATCTACGGT-3'	
A0W34_31420 (OX-1)-F	5'-ATCGGTACCTTACCGACCGCGGCGT-3'	
A0W34_31420 (OX-1)-R	5'-ATCGGATCCCCGTCACGATCGCTACC-3'	
A0W34_03910 (OX-2)-F	5'-ATCGGTACCCGGTCGAGGCCTTCGTC-3'	
A0W34_03910 (OX-2)-R	5'-ATCGGATCCATACGTAGCCATCGCTCCA-3'	
pET28a(+)-F1	5'-GTGATGATGATGATGATGGCTG-3'	Cloning of *jydB* gene using modified In-Fusion method
pET28a(+)-R1	5'-CACCACCACCACCACCACT-3'	
pET28a(+)-F2	5'-TGATGATGGCTGCTGCCCATG -3'	
pET28a(+)-R2	5'-TTTCGCACAGCACCACCACCACCACCACT-3'	
jydB-F1	5'-ATGACAACCACCAAGATCGAA-3'	
jydB-R1	5'-CTGTGCGAAACTGCCGAGGAA-3'	
jydB-F2	5'-TCATCATCACATG ACA ACCACCAAGATCGAA-3'	
jydB-R2	5'-CTGCCGAGGAATCGTTGCAAC-3'	

Table S2 Candidate genes encoding putative QQ enzymes in *Rhodococcus* sp. BH4

BH4 gene No.(name)	Gene annotation	Homologues	Identity (%)	QQ activity	Reference
A0W34_26705 (A1)	Hydrolase, alpha/beta fold family functionally coupled to Phosphoribulokinase	**AiiO** (Ochrobactrum sp. A44)	45	+	Czajkowski et al, 2011
A0W34_24065 (A2-1)	6-aminohexanoate hydrolase	**QsdB** (uncultured proteobacterium 90H6)	39	-	Tannieres et al, 2013
A0W34_00835 (A2-2)	Amidase	**QsdB** (uncultured proteobacterium 90H6)	36	-	
A0W34_31420 (OX-1)	Short-chain dehydrogenase	**BpiB09** (uncultured bacterium Bio5)	36	-	Bijtenhoorn et al, 2011
A0W34_03910 (OX-2)	Oxidoreductase		35	-	

Fig. S1. Construction of recombinant plasmids carrying the putative QQ genes of *Rhodococcus* sp. BH4. Candidate quorum quenching genes were amplified by PCR using Ex Taq DNA polymerase and primers designed to anneal 100bp upstream and downstream of the coding regions of the putative genes (Table S1). PCR cycling parameters were as follows; 95℃ for 20 s, 60℃ for 30 s, and 72℃ for 1 min, for 32 cycles. Purified amplicons were cloned into vectors (pMD20) and transformed into *E. coli* DH5α. Transformants were used for the AHL inactivation assay to assess activity of candidate QQ genes

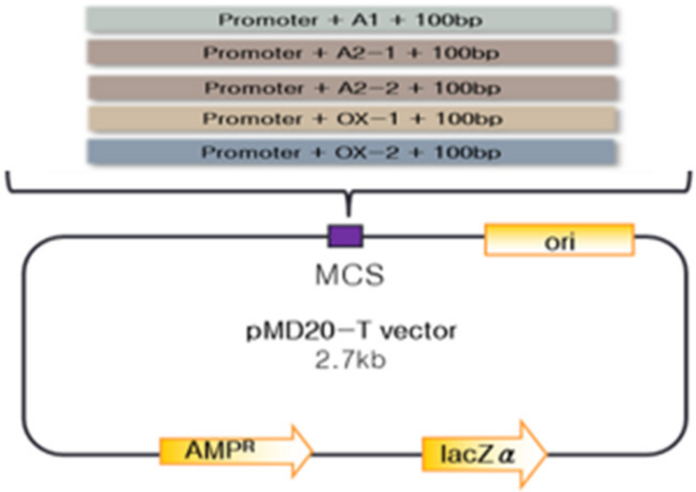

Fig. S2. SDS-PAGE analysis of purified recombinant His-tagged JydB from *E. coli* BL21(DE3). The protein was purified using an Ni-NTA column. Left lane (M) – molecular weight marker. Right lane – purified JydB. The size of the purified N and C-terminal 6XHis-tagged JydB protein was estimated at approximately 31 kDa.

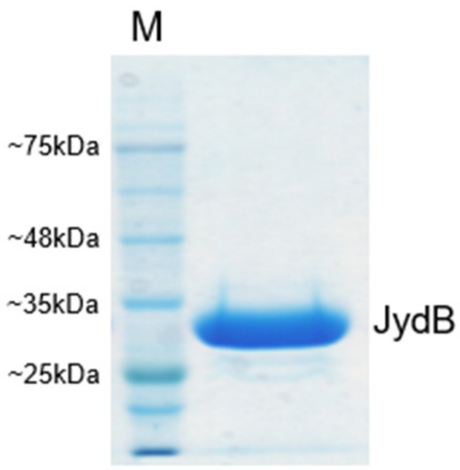

Fig. S3. AHL-degradation activity of purified JydB protein. Enzyme was mixed with C6-HSL, 3-oxo-C6-HSL and C8-HSL (final conc. 20 μM, 5 μM, and 1 μM, respectively) and incubated at 37℃ for 30min. Residual C6-HSL, 3-oxo-C6-HSL and C8-HSL are seen as halos on biosensor overlaid agar plates. N, negative control, samples with known concentrations of synthetic AHLs were used as control. J, reaction mixture of recombinant JydB and AHL.

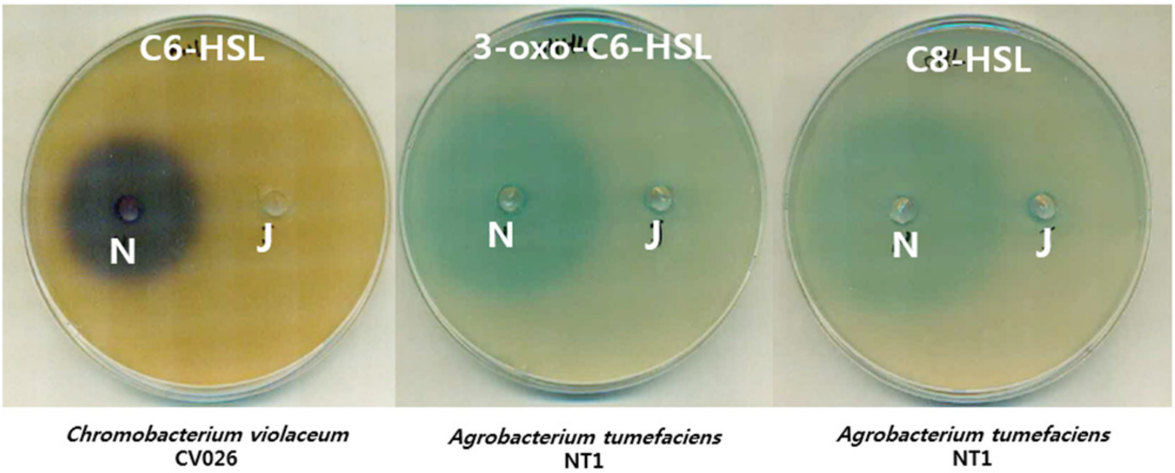

Supplement: Supplementary file 1 [file JMB-30-6-937-supple.pdf]
